# Tonic-Clonic Activity at Subarachnoid Hemorrhage Onset: Impact on Complications and Outcome

**DOI:** 10.1371/journal.pone.0071405

**Published:** 2013-08-12

**Authors:** Gian Marco De Marchis, Deborah Pugin, Hector Lantigua, Christopher Zammit, Prasanna Tadi, J. Michael Schmidt, M. Cristina Falo, Sachin Agarwal, Stephan A. Mayer, Jan Claassen

**Affiliations:** Departments of Neurology and Neurosurgery, Columbia University College of Physicians and Surgeons, New York, New York, United States of America; University of Cambridge, United Kingdom

## Abstract

**Objective:**

Tonic-clonic activity (TCA) at onset complicates 3% to 21% of cases of subarachnoid hemorrhage (SAH). The impact of onset TCA on in-hospital complications, including seizures, remains unclear. One study associated onset TCA with poor clinical outcome at 6 weeks after SAH, but to our knowledge no other studies have confirmed this relationship. This study aims to assess the impact of onset TCA on in-hospital complications, poor functional outcome, mortality, and epilepsy at 3 months.

**Methods:**

Analysis of a prospective study cohort of 1479 SAH patients admitted to Columbia University Medical Center between 1996 and 2012. TCA within 6 hours of hemorrhage onset was identified based on accounts of emergency care providers or family witnesses.

**Results:**

TCA at onset was described in 170 patients (11%). Patients with onset TCA were younger (P = 0.002), presented more often with poor clinical grade (55% vs. 26%, P<0.001) and had larger amounts of cisternal, intraventricular, and intracerebral blood than those without onset TCA (all, P<0.001). After adjusting for known confounders, onset TCA was significantly associated with in-hospital seizures (OR 3.80, 95%-CI: 2.43–5.96, P<0.001), in-hospital pneumonia (OR 1.56, 95%-CI: 1.06–2.31, p = 0.02), and delayed cerebral ischemia (OR 1.77, 95%-CI: 1.21–2.58, P = 0.003). At 3 months, however, onset TCA was not associated with poor functional outcome, mortality, and epilepsy after adjusting for age, admission clinical grade, and cisternal blood volume.

**Conclusions:**

Onset TCA is not a rare event as it complicates 11% of cases of SAH. New and clinically relevant findings are the association of onset TCA with in-hospital seizures, pneumonia and delayed cerebral ischemia. Despite the increased risk of in-hospital complications, onset TCA is not associated with disability, mortality, and epilepsy at 3 months.

## Introduction

Focal or generalized tonic-clonic activity (TCA) at the moment of subarachnoid hemorrhage (SAH) onset, or shortly following, has been described in 3% to 21% of patients.[1]–[7] TCA is often associated with loss of consciousness, and is described as varying combinations of stiffening or tonic posturing of the trunk and extremities and clonic twitching of the face or extremities. Although onset TCA presumably reflects seizure activity, the precise causes remain uncertain, since generalized tonic posturing and clonus can also occur as a manifestation of corticospinal tract injury due to acute intracranial hypertension and brain stem herniation. Even though onset TCA is reported in up to 1 out 5 SAH patients, it remains unclear whether onset TCA is associated with in-hospital complications, including in-hospital seizures. Regarding prognostic relevance, one study associated onset TCA with poor clinical outcome after SAH at 6 weeks and recurrent seizures [2], but to our knowledge no other studies have confirmed these relationships. In this study, we sought to investigate whether onset TCA is associated with in-hospital seizures and other prespecified complications, including mortality, poor functional outcome, and epilepsy 3 months after SAH.

## Methods

### Ethics Statement

The Institutional Review Board at Columbia University Medical Center approved the study protocol. Written informed consent was obtained at the time of admission from patients or, if neurologically impaired, from family members.

### Patients

All patients with SAH admitted to the Columbia University Medical Center Neurological Intensive Care Unit between July 1996 and September 2012 were offered enrollment in the Columbia University SAH Outcomes Project. At the time of admission, clinical events at onset were recorded by interviewing eyewitness prehospital and emergency department care providers and family members.

### Clinical Definitions and Clinical Management

Onset TCA was defined as focal or generalized tonic stiffening or posturing, rhythmic clonic or myoclonic jerking movements, or both, of the face or extremities with or without loss of consciousness occurring within 6 hours of the initial bleeding event. Upon admission, the treating team determined occurrence of onset TCA based on accounts from witnesses of SAH onset. In-hospital seizures were defined as either witnessed focal or generalized tonic-clonic seizures or non-convulsive seizures confirmed by continuous electroencephalography. Clinical grade was assessed after adequate resuscitation upon admission to the Columbia University Medical Center with the Hunt-Hess Scale [8], with grades 4 and 5 defined as poor grade. Admission CT scans were prospectively evaluated using the modified Fisher Scale (mFS) [9] and Hijdra SAH sum score [10]; the presence and volume of intraparenchymal hemorrhage using the ABC/2 method [11]; and volume of intraventricular hemorrhage (IVH) using the IVH sum score (range 0–12 points). [12] Pneumonia was defined as new infiltrate on chest X-ray with fever or purulent sputum. [13] The time of onset of pneumonia was not recorded. Rebleeding was defined as acute, in-hospital worsening in neurologic status along with an increase in hemorrhage volume on a repeat CT scan. [14] Delayed cerebral ischemia (DCI) was defined as either delayed ischemic neurological deterioration, a new infarct on CT scan, or both, attributed to vasospasm by consensus review of the study team in weekly meetings. [15] Systemic inflammatory response syndrome (SIRS) was included to explore the hypothesis that onset TCA may be linked to SIRS. Features of SIRS were scored by summing the number of variables meeting prespecified criteria (HR >90/min, RR >20/min, Temperature >38°C or <36°C, WBC count <4,000 G/L or >12,000 G/L). SIRS burden over the first four hospitalization days was calculated from the mean of daily SIRS scores. [16].

All patients received antiepileptic drugs (AEDs) on admission. In general, AEDs (phenytoin or levetiracetam) were continued for a minimum of 3 months in patients with onset TCA or documented in-hospital seizures, for 7–14 days in poor grade patients even in the absence of seizures, and were discontinued on post-operative day 1 in good grade patients without seizures. Medical and surgical management was otherwise performed as previously described. [17] Trained personnel assessed outcome using the modified Rankin scale (mRs) and seizure recurrence by in-person interview or telephone interview at 3 and 12 months. Favorable functional outcome was defined as mRs of 0 to 3, unfavorable functional outcome as mRs of 4 to 6. Outcome at 3 months constituted the primary outcome of interest for the present study.

### Statistical Analysis

Categorical variables were compared with the Fisher’s exact test and continuous variables with the Mann-Whitney *U* test. Number needed to harm was calculated as 1/(absolute risk difference). We performed 5 multivariate logistic regression analyses to assess the association between onset TCA and the following outcome variables: 1) in-hospital seizures; 2) in-hospital pneumonia; 3) DCI; 4) unfavorable functional recovery at 3-months (mRs 4–6); and 5) mortality at three months. In all logistic regression models, we included poor clinical grade on admission - because of its established prognostic relevance – and additional covariates known to be associated with the outcome of interest. SIRS burden was available in a subset of patients (n = 474), where we ran a linear regression model with SIRS as the outcome variable with onset TCA and other reported SIRS predictors as covariates. [16] Type-1 error was preset at 0.05, so that a *P* value of ≤0.05 was considered statistically significant. All statistical calculations were made with *Stata, Version 12*.

## Results

### Baseline Characteristics

Between 1996 and 2012, we enrolled 1479 patients, of whom 170 (11%) had a report of onset TCA. Patients with TCA were younger; presented more often with ictal loss of consciousness and with poor clinical grade; had larger volumes of cisternal and ventricular blood; and more often had intraparenchymal hemorrhage on admission CT. We observed no difference in gender, race/ethnicity, past medical history of epilepsy, antiepileptic drug use or aneurysm location and volume of intraparenchymal hemorrhage (Table 1).

**Table 1:** Baseline Characteristics in SAH Patients With and Without Onset TCA.

	Onset TCA		No Onset TCA		*P* Value
	(*N = 170*)		(*N = 1309*)		
Age, years	50	(41–60)	54	(45–64)	**0.002**
Women	116	(68.2)	872	(66.6)	0.93
Non-White Race/Ethnicity, n (%)	101	(59.4)	701	(53.6)	0.16
Epilepsy in Past Medical Hx	5	(2.9)	21	(1.6)	0.20
Antiepileptic Drugs in Past Medical Hx	4	(2.4)	21	(1.6)	0.52
Ictal Loss of Consciousness, n (%)	131	(77.1)	446	(34.1)	**<0.001**
Location of the Ruptured Aneurysm					
Anterior Circulation†	126	(74.1)	874	(66.8)	0.73
Middle Cerebral Artery	39	(22.9)	218	(16.6)	0.21
*Hunt & Hess Grade, n (%)*					
1, 2 or 3	77	(45.3)	964	(73.6)	**<0.001**
4 or 5	93	(54.7)	345	(26.4)	
*Modified Fisher Grade, n (%)*					
1, no thick cisternal blood, – IVH	32	(18.8)	492	(37.6)	**<0.001**
2, no thick cisternal blood,+IVH	17	(10.0)	109	(8.3)	
3, thick cisternal blood, – IVH	66	(38.8)	464	(35.5)	
4, thick cisternal blood,+IVH	55	(32.4)	244	(18.6)	
Hijdra SAH Sum Score*	19	(11–24)	14	(7–21)	**<0.001**
Intracerebral hemorrhage, n (%)	54	(31.8)	178	(13.6)	**<0.001**
Intracerebral hemorrhage volume (ml)	11.5	(5–24)	7	(3–22)	0.23
IVH Sum Score**	2	(0–5)	1	(0–3)	**<0.001**

Data are N (%) or median [IQR]. TCA denotes tonic-clonic activity at subarachnoid hemorrhage onset.

* Range 0, no blood, 30 all cisterns completely filled.

** Range 0, no IVH, 12 all ventricles completely filled with IVH.

† Anterior circulation denotes: anterior cerebral artery, anterior communicating artery, intracranial internal carotid artery, middle cerebral artery, posterior communicating artery.

### In-hospital Complications

In-hospital complications were more frequent in patients with TCA (Table 2).

**Table 2:** Complications, Length of In-hospital Stay and Outcomes According to Onset TCA.

	Onset TCA		No Onset TCA		*P* Value
	(*N = 170*)		(*N = 1309*)		
Mean SIRS score, first 4 days*	2.6	(2.2–3.2)	2.0	(1.5–2.6)	**<0.001**
*Neurological Complications*					
In-hospital Seizures, n (%)	38	(22.4)	76	(5.8)	**<0.001**
Delayed Cerebral Ischemia**					
Ø Symptoms/ Ø Infarction, n (%)	110	(64.7)	1002	(76.6)	**0.001**
+ Symptoms/ Ø Infarction, n (%)	25	(14.7)	121	(9.2)	
Ø Symptoms/+ Infarction, n (%)	6	(3.5)	37	(2.8)	
+ Symptoms/+ Infarction, n (%)	21	(12.4)	76	(5.8)	
Hydrocephalus (treated with EVD), n (%)	91	(53.5)	461	(35.2)	**<0.001**
Global Cerebral Edema	76	(44.7)	317	(24.2)	**<0.001**
Rebleeding	26	(15.3)	102	(7.8)	**0.002**
Pneumonia, n (%)	60	(35.3)	244	(18.6)	**<0.001**
*Length of Stay*					
In the Neurocritical Care Unit (days), median (IQR)	12	(7–18)	8	(5–13)	**<0.001**
In the Hospital (days), median (IQR)	17	(9–26)	12	(9–20)	**<0.001**
*Outcome at 3 months*					
Seizures after discharge	1	(0.6)	35	(2.7)	0.25
Poor functional outcome (mRs 4 or 6)	60	(35.3)	307	(23.4)	**0.001**
Death (mRs 6)	43	(25.3)	236	(18.0)	**0.02**

Data are N (%). TCA denotes tonic-clonic activity at subarachnoid hemorrhage onset.

* SIRS denotes systemic inflammatory response, with range 0 lowest, 4 highest.

** Information on DCI was not available for 81 patients.

#### In-hospital seizures

A total of 114 (8%) patients had in-hospital seizures. Patients with TCA were almost 4 times more likely to develop in-hospital seizures (22% vs. 6%, P<0.001). The association between TCA and in-hospital seizures remained significant after adjusting for two variables associated with in-hospital seizures (rebleeding and large cisternal blood volume) and poor admission clinical grade (adjusted odds ratio [OR] 3.80, 95%-CI: 2.43–5.96, P<0.001) (Table 3). Even after additional adjusting for aneurysm clipping and acute hydrocephalus, the association between TCA and in-hospital seizure remained significant (adjusted OR 3.70, 95%-CI: 2.34–5.83, P<0.001).

**Table 3:** Multivariate Analysis.

	OR	95%-CI		P Value
*Prediction of In-hospital Seizures:*				
Onset TCA	3.80	2.43	−5.96	**<0.001**
Hunt & Hess 4 or 5	1.50	0.98	−2.30	**0.06**
Rebleeding	2.65	1.58	−4.47	**<0.001**
Modified Fisher Score 3 or 4	1.28	0.83	−1.97	0.27
Prediction of In-hospital Pneumonia:				
Onset TCA	1.56	1.06	−2.31	**0.02**
Hunt & Hess 4 or 5	4.11	3.04	−5.57	**<0.001**
Age (1 year increase)	1.02	1.01	−1.03	**<0.001**
Ictal loss of consciousness	1.64	1.21	−2.24	**0.002**
*Prediction of Delayed Cerebral Injury:*				
Onset TCA	1.77	1.21	−2.58	**0.003**
Hunt & Hess 4 or 5	1.99	1.49	−2.66	**<0.001**
Modified Fisher Score 3 or 4	1.52	1.14	−2.04	**<0.001**
Smoking (ever)	1.32	1.01	−1.74	**0.04**
*Prediction of Unfavorable Functional Outcome at 3 months* *				
Onset TCA	1.30	0.81	−2.09	0.28
Hunt & Hess 4 or 5	14.03	10.01	−19.65	**<0.001**
Age (1 year increase)	1.05	1.04	−1.06	**<0.001**
Modified Fisher Score 3 or 4	1.48	1.06	−2.06	**0.02**
*Prediction of Mortality at 3 months*				
Onset TCA	0.97	0.60	−1.56	0.90
Hunt & Hess 4 or 5	12.14	8.66	−17.02	**<0.001**
Age (1 year increase)	1.04	1.03	−1.05	**<0.001**
Modified Fisher Score 3 or 4	1.39	0.98	−1.98	0.07

* Unfavorable functional outcome defined as mRs of 4, 5 or 6.

#### In-hospital pneumonia

A total of 304 (21%) patients developed in-hospital pneumonia. Patients with onset TCA developed significantly more often pneumonia (35% vs. 19%, p<0.001). The association between TCA and pneumonia remained significant even after adjustment for poor admission clinical grade, age, and ictal loss of consciousness (adjusted OR 1.56, 95%-CI: 1.06–2.31, p = 0.02).

#### Delayed cerebral ischemia

**Figure 1:**
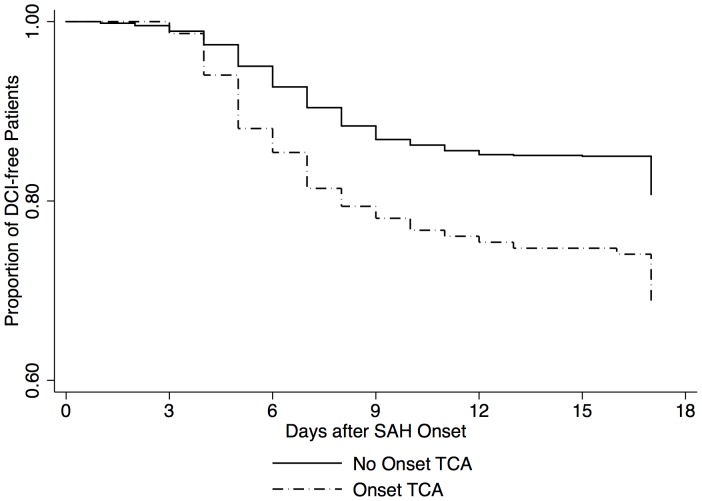
DCI denotes delayed cerebral injury, TCA tonic-clonic activity.

Patients with onset TCA more frequently developed DCI and notably more symptomatic infarcts (12% vs. 6%, p<0.001). The time-to-DCI Kaplan-Meyer curves differed significantly, with higher rate of DCI in the group with TCA (Fig. 1). The association between TCA and DCI remained significant even after adjustment for poor clinical grade on admission, large cisternal blood volume, and smoking [18] (adjusted OR 1.77, 95%-CI: 1.21–2.58, P = 0.003) (Table 3). TCA was not associated with vasospasm visible in the admission cerebral angiography (10.0% vs. 7.6%, P = 0.5).

#### SIRS

Onset TCA was associated with higher SIRS burden over the first 4 hospital days (Table 2). On average, onset TCA was associated with a higher SIRS score by 0.27 points after adjusting for established SIRS predictors [16] including poor clinical grade, thick cisternal blood, aneurysm size, aneurysm clipping, hydrocephalus, and in-hospital pneumonia (adjusted Coefficient for SIRS = 0.27, 95%-CI: 0.05–0.50, P = 0.02).

### Outcome

At 3 months, poor functional outcome and mortality were more common among patients with onset TCA (Table 2). However, these associations did not remain significant after adjusting for poor admission clinical grade, age, and cisternal hemorrhage volume (Table 3). We observed no significant association between onset TCA and seizures within 3 months from hospital discharge (Table 1). Similarly, at 12 months, no association of onset TCA with poor functional outcome, mortality, and epilepsy was noted (data not shown).

## Discussion

TCA was present in 11% of SAH patients and was more common among patients of younger age and those with larger blood volumes in their basal cisterns, ventricles, and within their brain parenchyma. The novel, clinically relevant findings were that patients presenting with onset TCA had a 3.8 times and 1.56 times higher adjusted odds of developing in-hospital seizure and pneumonia, respectively. Onset TCA did not significantly predict either functional outcome or mortality at three months after adjusting for known predictors. Despite the increased risk of in-hospital complications, onset TCA is not associated with disability, mortality, and epilepsy at 3 months – good news for patients and their families.

### Admission Findings

The observed frequency of onset TCA falls in the range reported by other studies (3%–21%).[1]–[7] The most important reason for such a wide prevalence range is likely the inaccuracy inherent in reporting onset TCA from bystanders. Another reason may lie in the differences of the time interval employed to categorize seizures as “onset TCA”, ranging from seizures occurring before hospital admission (“in the field”) [6] up to 24 hours from SAH onset. [19] To categorize seizures as onset TCA, we chose a uniform time interval of 6 hours from SAH onset because time from SAH onset to hospital admission may vary across patients. While every definition is ultimately arbitrary, the choice of a 6-hour time interval seems supported by a study from the Royal Melbourne Hospital in Australia, whereby all onset TCA occurred within the first 6 hours of the 24-hour time interval used to define onset TCA. [2].

Age seems to matter, and the significantly younger age observed in patients with onset TCA is in line with the literature. The highest reported frequency of onset TCA (21%) was found in a study including 95 SAH patients aged between 15–45 years admitted to the University Hospitals of Iowa, US. [3] Similarly, 23% of patients aged under 31 experience onset TCA vs. only 4% of patients over age of 50 in the study from the Royal Melbourne Hospital center. [2] The association between onset TCA and young age is not entirely clear. The brain of younger individuals seems *per se* more susceptible to seizures (prevalence of excitatory circuits over inhibitory ones) [20] and early age represents an established risk factor for early seizures in traumatic brain injury. [21] The association between onset TCA and SAH severity is in line with the findings of a study from the Hospital Santa Maria in Lisbon Portugal, whereby onset TCA was associated with admission Hunt & Hess grades of 4 or 5 and with large volumes of subarachnoid blood. [5] The larger blood volumes in the basal cisterns and ventricles of patients with onset TCA are also in line with the study from the Royal Melbourne Hospital, which – however – did not observe an association with higher Hunt and Hess grade on admission. [2] Subarachnoid blood and the resulting products may have an epileptogenic activity [22], but this remains a speculative explanation.

### In-hospital Seizures

The observed association between ictal and in-hospital seizures is a clinically relevant finding because studies focusing on in-hospital seizures are scant. Moreover, the association of onset TCA with in-hospital seizure remained significant after adjusting for hydrocephalus and aneurysm clipping, known risk factors for in-hospital seizures. [19] Patients with onset TCA admitted to the Royal Melbourne Hospital had 27 higher odds of developing seizures within the first 6 weeks. [2] While the time interval of 6 weeks seemed to include both in-hospital seizures and seizures after discharge, it would be reasonable to think that such a strong association might hold true also for the subset of in-hospital seizures. On the other hand, patients with onset TCA admitted between 1955–78 to two hospitals in Missouri (US) did not show increased odds of in-hospital seizures. [7] This difference might be due to a power limit in the latter study (sample size: n = 100). Finally, in our cohort, the systematic use of prophylactic AED may have reduced the rate of in-hospital seizures.

### In-hospital Pneumonia

The association between onset TCA and pneumonia was strong (number needed to harm = 6, meaning: per 6 patients with onset TCA, 1 additional in-hospital pneumonia was observed), and remained significant even after adjusting for ictal loss of consciousness. Therefore, onset TCA seem to represent an additional risk factor for pneumonia. One possible reason is that patients with onset TCA are more prone to bronchoaspiration, even if we cannot prove this, as we did not differentiate between aspiration and non-aspiration pneumonia. Nevertheless, the association to in-hospital pneumonia is of clinical relevance, and likely contributed to patients with onset TCA staying 5 days longer in the hospital.

### Delayed Cerebral Ischemia

Given the small magnitude of association, the link between onset TCA and DCI is interesting, but should be viewed as hypothesis generating. For example, the highest absolute risk difference (6.6%) was observed in the DCI subcategory “symptomatic infarcts”, corresponding to a number needed to harm of 15. A hypothesis is that onset TCA aggravates SIRS, which in turn is associated with DCI (*hypothesis*: onset TCA SIRS DCI). [16] We found that onset TCA was associated with higher SIRS burden over the first 4 hospitalization days even after adjusting for both known predictors of SIRS as well as hydrocephalus and in-hospital pneumonia. This finding is in line with the increasing body of evidence suggesting that seizures can actually cause inflammation. [23] The association between onset TCA and DCI remained significant also after adjusting for SIRS, suggesting that additional mechanisms other than SIRS link onset TCA to DCI. Alternatively, cortical spreading depression may link onset TCA with DCI, but the available data do not allow us to explore this relationship. [24] It is prudent to remember that both SIRS and cortical spreading depression are not as established predictors of DCI as poor clinical grade and large volume of cisternal blood.

### Functional Outcome, Mortality and Seizures at 3 Months

At hospital discharge, in 256 SAH patients from Hospital Santa Maria in Lisbon (Portugal), onset TCA was associated to a mRs ≥4 (OR 4.1, 95%-CI 1.4–11.6, P<0.05), but a multivariate analysis is not available. [5] At 6 weeks, onset TCA was associated with disability after adjusting for known outcome predictors (OR 7.8; 95%-CI: 1.1–13.9, p = 0.04) in the study from the Royal Melbourne Hospital in Australia. [2] At 3 months, we did not observe any association between onset TCA and both functional outcome and mortality after adjustment for known outcome predictors. The clinical relevance of this finding is that onset TCA, despite being associated with in-hospital complications, does not represent a negative prognostic finding by itself. It is unclear whether the longer time of AED administration in patients with onset TCA - three months instead of only 2 weeks - improved outcomes in patients with TCA. Similarly to outcome, the association between onset TCA and late seizures seems to fade off over time: within 6 weeks, onset TCA was associated with late seizure (OR 27.4, 95%-CI: 2.3–330, p<0;01). [2] Within 12 months, onset TCA was not associated with late seizure/epilepsy. [4] No association between onset and late seizure was observed in a study from the University Hospital Rotterdam Dijkzigt (The Netherlands), whereby the follow-up for late seizures spanned up to 4.8 years. [1].

### Limitations

This study has limitations. Both onset TCA and in-hospital seizures are clinical syndromes and as such they do not allow inferring the underlying etiology (epileptic activity vs. symptoms from elevated intracranial pressure). However, this study aimed at looking at the consequences of onset TCA, rather than exploring their causes. Second, timing of in-hospital seizure was not available, and information on SIRS was available only in the last enrolled 474 patients. Finally, sufficiently detailed information on AED at or after hospital discharge was not available.

### Conclusion

This study shows that onset TCA is not a rare event, as it occurs in more than 1 out of 10 patients with SAH. New findings are that onset TCA is associated with an increased risk of in-hospital seizures, pneumonia and DCI. Given the association with in-hospital seizure, AED might be considered in SAH patients with onset TCA. Use of AED might be considered in SAH patients with onset TCA. However, only a randomized clinical trial can ultimately answer the question whether AED improve outcome after SAH. Despite the increased risk of in-hospital complications, onset TCA is not associated with disability, mortality, and epilepsy at 3 months. These observations add to the literature as prior studies report an association between onset TCA and unfavorable outcome at 6 weeks. The lack of association with outcome at 3 months is important: despite an increased risk of in-hospital complications, onset TCA should not encourage withdrawal of care among patients with SAH.
